# Evaluation and Validation of a Real-Time Polymerase Chain Reaction Assay for Rapid Identification of Bacillus anthracis Supplement

**DOI:** 10.3201/eid0810.021001

**Published:** 2002-10

During the recent outbreak of bioterrorism-associated anthrax in the United States, 11 patients were diagnosed with inhalational anthrax and 7 with cutaneous anthrax (Table 1 and 2) (1–6). During the extensive epidemiologic investigation, >125,000 clinical and environmental specimens were collected and analyzed for Bacillus anthracis, the causative agent of anthrax. We used the Laboratory Response Network (LRN) polymerase chain reaction (PCR) assay (real-time PCR assay) during the anthrax outbreak to detect B. anthracis DNA in environmental samples and clinical specimens. This assay provided 100% sensitivity and specificity when evaluated and validated on our panel of diverse bacterial isolates. On clinical specimens, this assay was one of three used to confirm anthrax cases when isolation of B. anthracis failed after antimicrobial drug treatment was initiated. In these culture-negative cases, laboratory confirmation was based on at least two supportive laboratory tests including this PCR, immunohistochemical stain (IHC), or anti-protective antigen (PA) titer (immunoglobulin [Ig]G enzyme-linked immunosorbent assay [ELISA]). PCR assays have not been previously used in an outbreak setting to detect B. anthracis directly in clinical specimens in a real-time manner. We evaluated the use of this assay on the clinical specimens and environmental samples received during the outbreak.

**Table 1:** Laboratory methods used for confirmation of 11 inhalational anthrax cases^a,b^

Patient no.^c^	Laboratory confirmation	Other laboratory tests positive for Bacillus anthracis
1	CSF culture	IHC of multiple (postmortem) tissues, blood culture
2	PCR of pleural fluid; IHC of pleural fluid; serology	Transbronchial biopsy IHC, pleural biopsy IHC
3	Blood culture	PCR of blood; serology
4	Blood culture	Serology
5	Blood culture	IHC of mediastinal lymph nodes; PCR of blood
6	Blood culture	IHC of mediastinal lymph nodes; PCR of blood
7	Blood culture	Serology
8	PCR of pleural fluid; IHC of pleural fluid	Serology
9	IHC of pleural fluid and bronchial biopsy; serology	
10	Blood and pleural fluid culture	IHC of multiple organs; PCR of multiple organs
11	Blood culture	PCR of multiple organs; IHC of multiple organs

^a^All initial isolation of Bacillus anthracis from clinical specimens took place at the local health facility where the patients were treated. ^b^CSF, cerebrospinal fluid; IHC, immunohistochemical stain; PCR, polymerase chain reaction. ^c^Patients 1–10 described in Jernigan et al. (1) and patient 11 in Barakat et al. (5).

**Table 2:** Laboratory methods used for confirmation of seven cutaneous anthrax cases^a,b^

Patient no.	Laboratory confirmation
1	Chest biopsy IHC, serology
2	Arm biopsy IHC and PCR, serology
3	Arm biopsy IHC, serum PCR
4	Face biopsy IHC, serology
5	Blood culture
6	Forehead biopsy IHC and PCR
7	Face biopsy culture

^a^All initial isolation of Bacillus anthracis from clinical specimens took place at the local health facility where the patients were treated. ^b^IHC, immunohistochemical stain; PCR, polymerase chain reaction.

Real-time PCR performance was evaluated by using clinical specimens collected from the nine confirmed cases of inhalational anthrax and seven confirmed cases of cutaneous anthrax identified during the bioterrorism-associated anthrax outbreak from October to December 2001. An effort was made to obtain the exact time of collection for each clinical specimen; however, when these data were not available, estimates were made based on other evidence from the medical record. A confirmed case of anthrax was defined as a clinically compatible case of cutaneous or inhalational illness that was either 1) laboratory confirmed by isolation of B. anthracis from an affected tissue or site or 2) accompanied with other laboratory evidence of B. anthracis infection based on at least two supportive laboratory tests, including (a) evidence of B. anthracis DNA by PCR from specimens collected from an affected tissue or site, (b) demonstration of B. anthracis in a clinical specimen by IHC, or (c) fourfold rise in anti-PA IgG. Further testing will be necessary for full evaluation of the utility of these methods on clinical samples. However, as more specimens became available, the LRN PCR was used as part of the laboratory confirmation of anthrax in this outbreak setting. Although real-time PCR results were part of initial confirmation of the diagnosis in 2 patients, all 18 patients were subsequently found to have sufficient laboratory evidence (i.e., culture, serologic testing, or IHC) to confirm case status without considering real-time PCR assay results (Table 1 and 2). In addition, 14 of 18 patients had sufficient laboratory evidence (i.e., real-time PCR, serologic testing, and IHC) to confirm case status without considering culture results (Table 1 and 2).

During the course of the outbreak investigation, clinical specimens were available from 74 patients who had initial symptoms similar to those of anthrax, but in whom the diagnosis was excluded after further evaluation. The exclusion of the diagnosis in these patients was based on the following: 1) the subsequent clinical course was not consistent with anthrax, 2) no laboratory evidence of B. anthracis infection was found, and 3) patient had sufficient negative laboratory evidence to establish that the confirmed-case definition could not be met (i.e., negative culture results or negative results on at least two other supportive laboratory tests).

The clinical performance of real-time PCR on clinical specimens was evaluated by using two approaches. In the first approach, traditional culture methods were used as the standard for evaluating real-time PCR detection of B. anthracis DNA in clinical specimens. In the second approach, the confirmed-case definition was used as the standard for comparing real-time PCR and traditional culture methods as diagnostic tests for anthrax.

A total of 279 clinical specimens were tested in parallel fashion by both traditional culture methods and by real-time PCR (Table 3). Two aliquots were prepared from each specimen. From one aliquot DNA was extracted with a MagNa Pure LC instrument (Roche Diagnostics GmbH, Mannheim, Germany) by employing a DNA isolation kit I with the “High Performance” protocol. In addition, select specimens were extracted in duplicate with a Qiagen DNA Mini Kit (Qiagen, Valencia, CA) per manufacturer’s instructions. A second aliquot was used to inoculate bacteriological media for isolation of B. anthracis (7).

**Table 3:** Results of real-time PCR and culture testing performed on 382 clinical specimens^a,b^

	PCR only		PCR and culture				Total
Nine inhalational cases	+	-	PCR + C -	PCR + C +	PCR - C -	PCR - C +	
Blood specimens	5	20	9	5	35	0	74
Swab specimens	0	0	0	0	0	0	0
Serum specimens	2	15	3	0	16	0	36
Sputum specimens	0	1	1	0	0	0	2
Tissue specimens	0	1	3	0	3	0	7
Pleural fluid							
specimens	5	0	11	0	3	0	19
Other specimens	1	0	2	0	1	0	4
Totals	50	92	142				
Seven cutaneous cases							
Blood specimens	0	0	1	0	10	0	11
Swab specimens	0	0	0	0	3	0	3
Serum specimens	0	2	0	0	11	0	13
Sputum specimens	0	0	0	0	0	0	0
Tissue specimens	0	2	2	0	4	0	8
Other specimens	0	0	0	0	2	0	2
Totals	4	33	37				
Four suspect cases							
Blood specimens	0	2	0	0	7	0	9
Swab specimens	0	0	0	0	2	0	2
Serum specimens	0	1	0	0	3	0	4
Sputum specimens	0	0	0	0	0	0	0
Tissue specimens	0	2	0	0	0	0	2
Other specimens	0	0	0	0	0	0	0
Totals	5	12	17				
Other							
Blood specimens	0	16	0	0	58	0	74
Swab specimens	0	1	0	0	14	0	15
Serum specimens	0	11	0	0	30	0	41
Sputum specimens	0	0	0	0	4	0	4
Tissue specimens	0	14	0	0	24	0	38
Other specimens	0	2	0	0	12	0	14
Totals	44	142	186				

^a^Sixteen patients with laboratory-confirmed anthrax, four suspect cases of anthrax, and 74 patients on whom anthrax has been ruled out. ^b^PCR, polymerase chain reaction; C, culture.

Specimens from patients meeting the definition for confirmed anthrax and from those in whom the diagnosis was excluded were tested by LRN PCR assay and traditional culture using the methods described above. For specimens that were unavailable for testing at Centers for Disease Control and Prevention (CDC), culture results reported by the clinical laboratories of the patient’s treating facility were used for case confirmation.

The performance of the LRN PCR assay was compared to that of traditional culture methods by testing environmental specimens collected from throughout the United States during the course of the outbreak by both methods. B. anthracis spores were eluted from swab specimens and other environmental samples in 2.5% pluronic F-68 (Sigma, St. Louis, MO) and then collected by centrifugation through an Ultrafree-CL, 0.45 uM, PVDF membrane filter (Millipore, Bedford, MA). Spores were eluted from the filters with 2.5% pluronic F-68, used to inoculate bacteriologic media, and added directly to real-time PCR assays without further purification or DNA extraction.

Two hundred seventy-nine clinical specimens were tested by both culture and real-time PCR: 92 were from 9 patients with inhalational anthrax, 33 from 7 patients with cutaneous anthrax, 12 from 4 patients with suspect cutaneous anthrax, and the remaining 142 from 74 patients in whom anthrax was excluded (Table 3). Of the 92 specimens from the inhalational anthrax cases, 5 (all blood specimens) were positive by both methods. Of the remaining 87, all were culture negative, but 29 (33%) were positive by the PCR assay. These included serum, sputum, pleural fluid, and tissue specimens (Table 3). Of the 33 specimens from the cutaneous anthrax cases, none were culture positive, but positive PCR results were obtained on a single blood specimen and two skin biopsy specimens. None of the 142 specimens from 74 patients without anthrax had positive results on culture or PCR.

A total of 382 clinical specimens from 94 patients were tested by real-time PCR, culture, or both. Real-time PCR was performed on specimens from 14 patients with anthrax in whom the diagnosis could have been confirmed using non-PCR methods, including 9 inhalational anthrax patients and 5 cutaneous patients (2 were confirmed by culture, 3 by IHC and serology, and 2 by IHC and PCR). PCR was also performed on specimens from 74 patients in whom anthrax was excluded.

Culture was performed on specimens from 13 anthrax patients in whom the diagnosis could be confirmed using non-culture methods, including 8 patients with inhalational anthrax and 5 patients with cutaneous anthrax. Culture was also performed on specimens from 74 patients in whom anthrax was excluded.

One hundred forty-two specimens tested in the patients with inhalational anthrax included blood (n=74), serum (n=36), sputum (n=2), tissue (n=7), pleural fluid (n=19), and other (n=4). One hundred eighty-six specimens tested in the patients without anthrax included blood (n=74), swabs (n=15), serum (n=41), sputum (n=4), tissue (n=38), and other (n=14).

## Inhalational Cases

Of the 11 patients with inhalational anthrax, 8 had blood cultures performed before the initiation of antimicrobial drug therapy, and cultures were positive in all eight at the hospital where patients were initially treated. At CDC, B. anthracis was also isolated from blood cultures of patient 5 (two blood cultures collected immediately before the start of the antimicrobial drug therapy), patient 6 (one blood culture collected on the same day antimicrobial drug therapy started),and patient 11 (two blood cultures collected the day before antimicrobial drug therapy). In contrast, 44 blood specimens were cultured from five patients (patients 2, 8, 9, 10, 11) (Table 1 and 2) after administration of antimicrobial drug therapy, and all were negative, including those from four patients (patients 2, 8, 10, 11) who had blood cultures obtained within 48 h of administration of antimicrobial drugs. Of the four patients (patients 2, 3, 5, 6) who had PCR performed on blood collected before the administration of antimicrobial drug therapy, all four had a positive PCR result (Table 4). In contrast, six patients had PCR testing of blood specimens collected after administration of antimicrobial drug therapy, and four (patients 2, 8, 10, 11) had a positive PCR result. A single patient (patient 2) had blood cultures collected >5 days after antimicrobial agent administration; a total of 26 blood specimens were collected past day 5, and 8 were PCR positive, ranging from day 7 to day 10 (Figure).

**Table 4:** Results of real-time PCR and culture testing performed on 142 clinical specimens collected from nine patients with inhalational anthrax^a^

		Antimicrobial drug therapy			
Patient no.^b^	Specimen type	Post therapy	Interval (days)	Culture	Real-time PCR
1	Pleural fluid^c^	Yes	3	Negative	Positive
	Serum^c^	Yes	3	Negative	Negative
	Serum^c^	Yes	3	Not done	Negative
	Pleural fluid^c^	Yes	4	Not done	Positive
	Pleural fluid^c^	Yes	4	Negative	Positive
	Blood^c^	Yes	4	Not done	Negative
	Right lung tissue (frozen)^c^	Yes	4	Not done	Negative
	Heart blood^c^	Yes	4	Not done	Negative
	Pericardial fluid^c^	Yes	4	Not done	Positive
2^c^	Blood (5)			Not done	Positive
	Blood (18)			Not done	Negative
	Blood (5)			Negative	Positive
	Blood (21)			Negative	Negative
	Serum (2)			Not done	Positive
	Serum (14)			Not done	Negative
	Serum (2)			Negative	Positive
	Serum (13)			Negative	Negative
	Pleural fluid (3)			Not done	Positive
	Pleural fluid (5)			Negative	Positive
	Pleural fluid (1)			Negative	Negative
	Body fluid (1)			Negative	Negative
	Respiratory wash (1)			Negative	Positive
	Sputum (1)			Not done	Negative
3	Blood culture	No	-1	Negative	Positive
5	Blood culture	No	0	Positive	Positive
	Blood culture	No	0	Positive	Positive
6	Blood culture	No	0	Positive	Positive
8	Blood	Yes	.5	Negative	Positive
	Blood	Yes	.5	Negative	Positive
	Serum	Yes	.5	Negative	Positive
	Serum	Yes	2	Negative	Negative
	Pleural fluid	Yes	2	Negative	Positive
	Pleural fluid	Yes	2	Negative	Positive
	Blood	Yes	37	Negative	Negative
9	Blood	Yes	2	Negative	Negative
	Blood	Yes	2	Negative	Negative
10	Pleural fluid	Yes	1	Negative	Positive
	Pleural fluid	Yes	1	Negative	Positive
	Blood	Yes	1	Negative	Positive
	Thioglycolate broth^c^	Yes	3	Negative	Negative
	CSF^c^	Yes	3	Negative	Positive
	Lung tissue (frozen)^c^	Yes	3	Negative	Positive
	Lymph node tissue (frozen)^c^	Yes	3	Negative	Positive
11	Blood culture	No	-1	Positive	Positive
	Blood culture	No	-1	Positive	Positive
	Sputum	Yes	2	Negative	Positive
	Body fluid/pleural fluid	Yes	2	Not done	Positive
	Blood	Yes	2	Negative	Negative
	Blood^c^	Yes	4	Negative	Negative
	Blood^c^	Yes	4	Negative	Negative
	Blood^c^	Yes	4	Negative	Negative
	Blood^c^	Yes	4	Negative	Negative
	Liver tissue (frozen)^c^	Yes	4	Negative	Negative
	Lymph node tissue (frozen)^c^	Yes	4	Negative	Positive
	Blood^c^	Yes	4	Negative	Negative
	Pleural fluid^c^	Yes	4	Negative	Negative
	Blood^c^	Yes	4	Negative	Negative
	Blood^c^	Yes	4	Negative	Negative
	Blood^c^	Yes	4	Negative	Negative
	Blood^c^	Yes	4	Negative	Negative
	Lung tissue (frozen)^c^	Yes	4	Negative	Negative
	Spleen tissue (frozen)^c^	Yes	4	Negative	Negative
	Blood^c^	Yes	4	Negative	Negative
	Pleural fluid^c^	Yes	4	Negative	Negative

^a^PCR, polymerase chain reaction  ^b^Patients 1–10 described in Jernigan et al*.* (1) and patient 11 in Barakat et al. (5). ^c^Samples collected postmortem. ^d^Due to the large number of samples from patient #2, samples were summarized by type and result in Figure.

**Figure:**
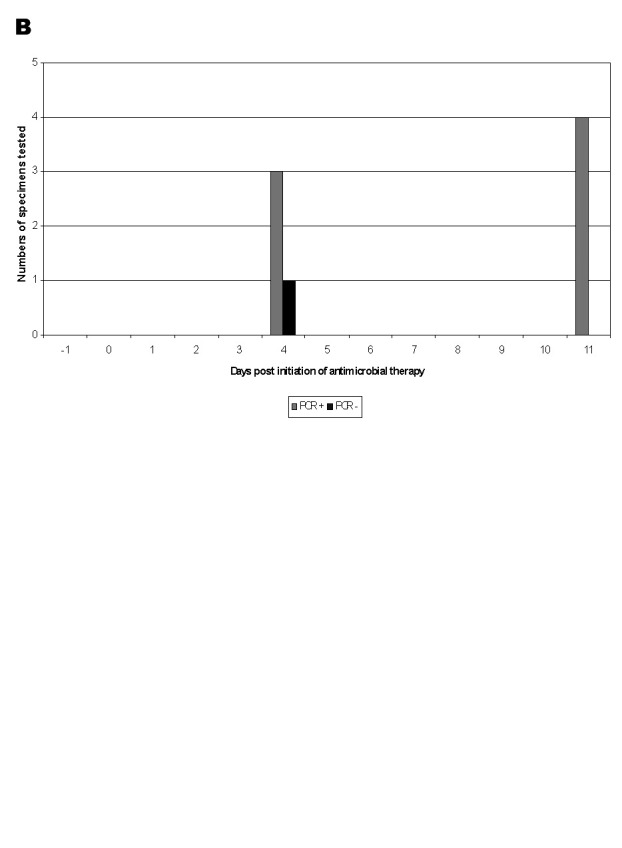
Results of polymerase chain reaction testing of clinical specimens (for which dates of collection were available) from a patient with inhalational anthrax (patient 2), are illustrated by date of collection relative to the initiation of antimicrobial drug therapy. Bacillus anthracis was not recovered from any of these specimens on which culture was attempted (data not shown). A. Blood, n=45; B. Pleural fluid, n=8.

Pleural fluid was available for testing from five patients (patients 1, 2, 8, 10, 11) with inhalational anthrax (Table 4). Of the pleural fluid specimens collected <5 days after the administration of antimicrobial drug therapy, none grew B. anthracis in culture, whereas all five had a positive PCR result. Pleural fluid specimens collected >5 days after antimicrobial agent administration were available only from a single patient (patient 2): all four of these specimens were PCR positive (Figure).

Seven postmortem tissue specimens were collected from three patients. Samples from a lymph node and lung tissue from one patient (patient 10) and a lymph node sample from another patient (patient 11) were PCR positive. All others were negative (Table 4).

Two sputum samples were tested. A sputum sample from patient 2 was received 5 days after the administration of antimicrobial drugs, and it was PCR negative. The second sputum was obtained on day 2 after the administration of antimicrobial drugs and was PCR positive (patient 11).

Of the seven patients with cutaneous anthrax, two had blood cultures performed before administration of antimicrobial drug therapy at the medical facility where patients were treated, and one patient had a positive result (Table 2, patient 5). All seven patients had blood cultures performed after initiation of antimicrobial drug therapy, and none had a positive result. All seven patients had PCR testing of blood specimens collected after administration of antimicrobial drug therapy, and of these, one was positive, from a patient with an extensive lesion and systemic complications of cutaneous anthrax. The blood sample was obtained 3 days after onset of the lesion. At the local facility, two patients had wound swabs obtained from ulcerative skin lesions before antimicrobial drugs were initiated; of these, one had evidence of gram-positive rods on Gram stain with B. anthracis isolated on culture (patient 7). Nine tissue samples were obtained from seven confirmed cases of cutaneous anthrax, including five patients with both fixed and frozen tissue and two patients with only fixed tissue. Eight samples were obtained after the administration of antimicrobial drugs. Culture was negative on all eight tissue samples; PCR was positive on one fixed tissue sample (patient 2), obtained 14 days after onset date, and on a fresh frozen tissue (patient 6) received 6 days after antimicrobial drugs were administered. In addition, one frozen tissue sample was received from a single patient before antimicrobial drug therapy; both culture and PCR were negative.Four additional cutaneous cases were defined as suspect because only one supportive laboratory test was positive; for three of the cases, serologic testing was positive, and for the fourth, IHC of an arm biopsy specimen was positive. A total of 12 specimens (7 blood specimens, 3 sera, 2 swabs) collected from these four patients were tested by PCR and culture; all were PCR and culture negative.

## Patients without Anthrax

One hundred eighty-six clinical specimens were collected from 74 patients who were subsequently determined not to have either inhalational or cutaneous anthrax; 142 specimens were culture and PCR negative (PCR specificity of 100%, 95% confidence interval 99% to 100%), and the remaining 44 tested by PCR only were also negative.

### Real-Time PCR in Environmental Specimens

One hundred forty environmental specimens were analyzed by both culture and real-time PCR. A wide variety of samples were tested, including dust, paper towels, a syringe, vent filters, HVAC filters, vacuum cleaner debris, a cellulose sponge, and clothing; however, most samples were surface swabs (n=82). Of the 140 environmental specimens tested by both PCR and culture, 35 were positive by both methods, 7 were positive by culture only, and 4 were positive by PCR only.

## Discussion

Isolation of B. anthracis from primarily sterile sites in culture has long been considered the standard of diagnosis for anthrax. However, this method is associated with a diagnostic delay of 12–24 h, and sensitivity is greatly diminished in the setting of prior antimicrobial administration (1). The LRN PCR was invaluable in diagnosing anthrax in patients when culturing B. anthracis failed and has rapidly become an integral part of the laboratory confirmation of anthrax. This real-time PCR also appears to be less affected by prior administration of antimicrobial drugs than culture, a property with important clinical ramifications. PCR positive results were obtained directly on clinical specimens, especially pleural fluids, in one case up to 11 days after the initiation of antimicrobial treatment.

When the LRN PCR and culture were simultaneously performed on clinical specimens, PCR was positive in every specimen from which B. anthracis was isolated. PCR was also positive in an additional 29 (33%) specimens that were culture negative. All PCR-positive specimens were collected from patients in whom the diagnosis of anthrax was confirmed by other methods, suggesting that LRN PCR has a higher positive predictive value than culture. The LRN PCR also appears to have high clinical specificity; no positive tests on clinical specimens were collected from patients in whom the diagnosis of anthrax was considered, but ultimately ruled out based on clinical course and additional diagnostic tests.

By using the confirmed case definition as the standard for diagnosis, the clinical performance characteristics of culture and LRN PCR during the 2001 outbreak can be directly compared to one another. Blood cultures appear to have a sensitivity of 100% (8 of 8 patients) if collected before the administration of antimicrobial drug therapy in patients with inhalational anthrax, but the sensitivity falls to zero if the blood is collected after administration of antimicrobial drugs. Similarly, PCR assay of blood has a sensitivity of 100% (6 of 6 patients) if the blood is collected before antimicrobial drug therapy. In contrast to blood culture, PCR assay can detect B. anthracis in the blood after administration of antimicrobial drug therapy. However, the sensitivity seems to decrease within 24 h after initiation of antimicrobial drugs; three of four inhalational anthrax patients who had PCR assay performed on blood collected within 24 h of antimicrobial administration had a positive result, while one of five patients who had PCR performed on blood collected >24 h after the start of antimicrobial drug therapy had a positive result.

The LRN PCR assay was particularly useful for testing pleural fluid specimens. No patient (n=5) in whom pleural fluid specimens were received at CDC had a culture positive result; however, all tests were performed after the administration of antimicrobial drug therapy. In contrast, all five patients who had the LRN PCR performed on pleural fluid specimens had a positive result including all three from whom pleural fluid was collected >24 hours after the administration of antimicrobial therapy. The sensitivity of the real-time LRN PCR on pleural fluid specimens appears to be less affected by the administration of antimicrobial drugs than does the LRN PCR of blood.

Laboratory confirmation for the seven cutaneous cases primarily relied on IHC and serology as only two clinical samples (one blood and one tissue sample) from two patients grew B. anthracis at the medical facility where the patients were examined and treated. However, the LRN PCR was subsequently attempted on 11 blood samples and 8 tissue samples from six cutaneous cases. Only one blood sample and two tissue samples from three patients were PCR positive (Table 3). CDC received all specimens from patients with cutaneous anthrax after the initiation of antimicrobial drug therapy. This success rate is similar to results of the LRN PCR on fluids (with the exception of pleural fluids) and tissue taken after the initiation of antimicrobial drug therapy on patients with inhalational anthrax.

Overall, B. anthracis was isolated from 8 (73%) of 11 patients with confirmed inhalational anthrax while the LRN PCR was positive for 8 (89%) of 9 patients tested. One case in which only two blood cultures were tested yielded negative results for both culture and PCR. Of the seven patients with confirmed cutaneous anthrax, B. anthracis was isolated from two patients (29%), and the LRN PCR was positive for three (43%).

One advantage of the LRN PCR assay is its rapidity; as a rule, results can be obtained within 1 h from the time samples have been prepared for testing. This rapid result is in striking contrast to the results for all other methods used for laboratory confirmation of anthrax. For example, standard culture methods require at least 24 h, while IHC results can be obtained within 8 h. On the other end of the spectrum is serology that requires paired sera collected at least 10 days apart, making this approach the least helpful in situations where therapeutic and public health decisions need to be instigated rapidly.

Evaluation of the LRN PCR and its performance on clinical specimens was not conducted as a true prospective study as we were, to a degree, limited by the number and type of specimens available, as well as by the emergent response needed to establish the microbiologic diagnosis. However, the number and variety of clinical samples were substantial enough to allow statistically significant comparisons with the current standard, culture. Also, the fact that laboratory confirmation was obtained by either culture or a combination of other supportive laboratory methods allowed for case-based evaluation of the LRN PCR’s sensitivity and specificity. A major advantage of the LRN PCR was its lack of any false-positive results (100% specificity) when used on cultures and directly on clinical specimens. Of the 110 patients clinically suspected to have anthrax, 74 had clinical samples collected and tested by at least three diagnostic approaches (culture, PCR, IHC, or serology) that would allow for a case to be defined as confirmed (culture positive or two supportive tests positive) or suspect (one supportive case positive). Samples from all of these patients were negative in all tests applied, including this LRN PCR. Given the extent and cost of public health and other actions taken after the laboratory confirmation of each anthrax cases in this epidemic, a false-positive PCR could have resulted in unnecessary waste of resources.

In addition to its invaluable use on clinical specimens, the LRN PCR also allowed for the rapid analysis of hundreds of diverse environmental samples throughout the outbreak investigation. If present in these specimens, B. anthracis was in the form of spores. Because B. anthracis spores contain DNA on their surfaces as a result of the sporulation process, environmental specimens can be analyzed in the PCR assay without having to do the DNA extraction, which eliminates the need for complicated and usually inefficient spore lysis methods. Culture methods and LRN PCR results were in agreement 92% (129/140) of the time. For the remaining 11 specimens, 4 were PCR positive and culture negative, and 7 were PCR negative and culture positive. The occasional discrepancies between culture and PCR could be due to inefficient removal of PCR inhibitors, detection of nonviable spores by PCR, and sampling error and volume effects when very few spores were present (5 µL for PCR vs. 100–200 µL for culture).

The LRN PCR assay evaluated and validated in this study detects a B. anthracis–specific chromosomal target as well as targets on both plasmids that are required for full virulence. This assay has served as an important aid in epidemiologic investigations of the recent bioterrorism-associated anthrax outbreak and was rapidly established as a valuable component of laboratory confirmation of anthrax cases. Highly specific results are obtained within a few hours of specimen arrival, making rapid and appropriate actions possible. At the same time, unnecessary panic and administration of antimicrobial drugs and vaccines were prevented when B. anthracis was rapidly excluded from differential diagnosis.
